# False Diplomas

**Published:** 1879-06

**Authors:** A. M. I. Hein


### ARTICLE III.

### *False Diplomas.*

Translated from Dr. A. Petermann's "Dental Almanac," by A. M. I. Hein, D. D. S.

In the first and second editions of the "Zahnarztlichen Almanach," I have given without respect to persons—for my combat only concerns the thing, never the person—several infallible proofs of the disreputable trade that was practised with the so-called American Dental Diplomas.

The evil continued to spread so rapidly amongst the dentists and quacks, (Kurpfuscher) that it was high time to stop the deceitful nuisance. Though it was known for years, that those purchasable diplomas were counterfeited, and not allowed by the law, the trade grew from year to year to larger dimension; the more so, because the possessors of those bought diplomas were not punished. The German Government did not know the magnitude of the infamous business.

The fact is, that besides barbers, surgeons, mechanics and others, dentists also adorn themselves with these fraudulent diplomas—and by no means to the honor of our profession.

Among those dentists are several having an esteemed position in society, and unfortunately they are members of the Central Association of German dentists, though they only can do harm to the reputation of the society.

For the dentists, the Diploma swindle is much more objectionable than in the case of the quacks, as the latter are without reputation, more or less on the ground of fraud and deceit.

To handle such improprieties and frauds among the dentists with velvet gloves, would only support the swindle. On the contrary, one ought to oppose with all possible means, such fraudulent pretentions, in order that the evil does not rise to gigantic dimensions.

Only by such means can we give to the dental profession, the respect it deserves, and not by distorting or concealing the whole truth.

The majority of the defrauders and those being cheated by false diplomas, have been quiet in real acknowledgement of their position, since the exposure in the first two editions of the Zahnarztlichen Almanach. It is so much more astonishing, when one of the Pseudo Doctors,—who has for years past, and until recently deceived his fellow-creatures, by using his bought and falsified Doctor———, title———, as he could know sufficiently, through German and foreign professional papers, that American Universities and Colleges, on account of their charters, could never confer a degree on persons *in absentia.* At last he sets himself on the high horse, and with clever distortion of the facts, allows himself, as if he was in full right, as Mr. Adolf Witzel, dentist in Essen an der Ruhr did, in the Oct. number, 1878 of the Deutschen [Vierteljahrsshrift] fur "Zahnheilkunde."

After I made it impossible by my exposures for this gentleman to use his bought Doctor title, he comes to the conclusion that he would have returned to the truth without my proceedings in stigmatizing the swindle.

It really shows great impudence if such people—who, themselves, dealt in bought and falsified Diplomas—as is the case with Mr. Adolf Witzel,—to write long discussions on Kurpfuscherei, mania for titles and means for the elevation of the honor of the dental profession.

It is not a true devotion for the science, nor interest for elevating the honor of the dental profession, that shuts their eyes against the rudest improprieties; personal respect, and exalted appreciation for the good of others should dictate a different course.

Such a ridiculous demand as Mr. Witzel makes reacts on himself. In the same discussion from what Mr. Witzel says, we find that he received such a Doctor's Diploma as he got, *in absentia,* but he makes it appear perfectly legal.

On the part of the German Reichs Kanzler, they have received through the Imperial Embassy in Washington, the terms for graduation of all the American Universities.

Alluding to the reports, now officially propagated, no American University has the privilege of giving any degree "*in absentia,*" etc. And if no University has the right to confer a degree "*in absentia,*" then this Doctor title of Mr. Witzel which he received "*in absentia,*" can not be legal, —his diploma is simply falsified and was purchased.

The Logic a la Witzel may be understood by those who can do so. Or does Mr. Witzel mean by legal that he paid for his Doctor's title?

If the discourse here is about doubtful American Universities, I need not say, that those who by honest work and true study "*in presentia*" in one of the reputable dental colleges, received the degree of Doctor of Dental Surgery, are free of all suspicion.

To the honor of the American Schools we are obliged to say, that none of them ever give a degree "*in absentia.*" Whoever wants to earn the title, has to make the trip to America.

Mr. Witzel says exactly, no college in America confers a degree, "*in absentia,*" only "*in præsentia,*" and only by honest work and study is the Doctor's degree obtained.

And anyhow he pretends his "*in absentia,*" bought and so-called American diploma to be real and legal.

It is impossible to understand that Mr. Witzel can have the insolence, contrary to his actions, to behave himself like a defender of the honor and dignity of the dental profession, and to write nonsense, like that referred to.

